# Block Copolymer Nanocomposites under Soft Confinement

**DOI:** 10.1021/acs.macromol.4c03184

**Published:** 2025-05-14

**Authors:** Javier Diaz, Marco Pinna, Andrei Zvelindovsky, Ignacio Pagonabarraga

**Affiliations:** † Departament de Física de la Matèria Condensada, Universitat de Barcelona, Martí i Franqués 1, Barcelona 08028, Spain; ‡ Universitat de Barcelona Institute of Complex Systems (UBICS), Universitat de Barcelona, Barcelona 08028, Spain; § School of Engineering and Physical Sciences, College of Health and Science, Center for Computational Physics, University of Lincoln, Brayford Pool, Lincoln LN6 7TS, U.K.

## Abstract

Block copolymer (BCP) melts can be blended with solvents to self-assemble into complex droplets with internal structures. Controlling the morphology of these softly confined structures is crucial for various applications, including drug delivery. The addition of nanoparticles (NPs) to BCP droplets produces hierarchical co-assembly with intricate structures, where BCPs act as scaffolds. However, incorporating NPs can significantly alter the BCP droplet structure, leading to emergent behavior. Computer simulations reveal that confinement-induced frustration leads to a Janus-like morphology, with spatially segregated hexagonal and lamellar structures within the droplet bulk. Systematic exploration of NP loading and chemical interactions demonstrates various phase transitions, which are rationalized based on changes in the effective composition and solubility of the BCP droplet. A time-dependent model enables the study of the kinetics of several NP-induced layered morphologies, indicating that changes in the effective solubility of the BCP droplet result in a slow progression toward an onion morphology.

## Graphical abstract

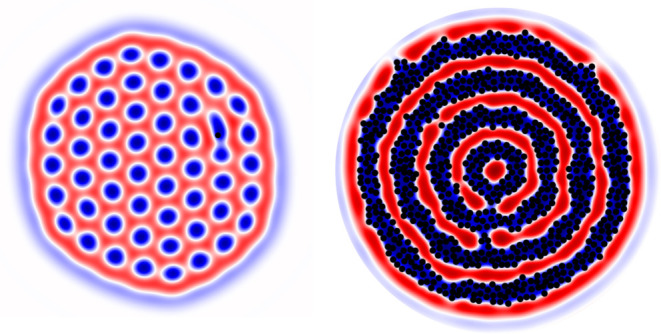

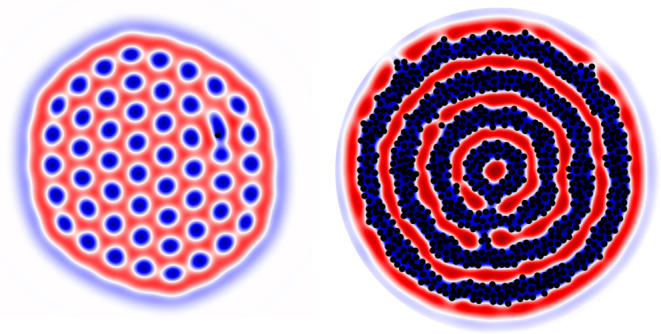

## Introduction

Materials composed of block copolymers (BCP) have received considerable attention due to various applications including lithography, nanopatterning, and nanoparticle (NP) design with applications in drug delivery;^1^ which stem from their self-assembly capabilities due to the heterogeneity of the BCP chain, leading to periodic structures with well-defined symmetry in the nanoscale. The morphology of BCP melts depends on the composition asymmetry and the degree of incompatibility between the BCP monomers.^2,3^ For diblock copolymers, this leads to a rich phase behavior including classical structures such as alternating lamellar, hexagonally ordered cylinders and body-centered-cubic (BCC) spheres, while more complex structures such as bicontinuous gyroid phases have been found.^4^

Beyond melts, BCPs have been blended with solvents such as homopolymers leading to ternary mixtures with a rich phase behavior.^5^ BCP dispersed in a solvent matrix can self-assemble into BCP-rich droplets with well-defined internal structure.^6,7^ Understanding the BCP droplet morphology under solvent-induced confinement is of crucial importance due to the various experimental and industrial applications involving NP design^8^ and drug delivery.^1,9^ These applications exploit the ability of BCP droplets to form spheroid objects with internal structure.^10^ The complex kinetic pathway connecting different stable BCP morphologies has been demonstrated in recent simulation works.^11,12^

Following the successful use of BCP melts as scaffolds to control the location of NPs^13^ NPs have been dispersed within BCP particles leading to complex and hierarchical structures. Early examples of NP segregation within BCP particles were used to obtain core-shell structures where the NP acted as a seed.^14^ Notable examples from Kim *et al* ^15–18^ used Au NPs in onion-like BCP droplets, where NPs could inherit the radial ordering of the BCP droplet. The shape of the hosting BCP particle can be greatly affected by the presence of NPs^19,20^ of different shapes.^21^ Shin reported that aggregation of Fe_3_O_4_ NPs can lead to morphological changes in the BCP particle, compared to Au NPs. Beyond spheroid particles, NPs can be distributed within cylindrical droplets.^22^ These recent experimental results involving BCP/NP hybrid particles suggest the possibility of designing and using new nanocomposite-based structures.^23,24^

Despite the large literature reporting theoretical or computational studies devoted to BCP nanocomposites in melt form^25^ there is comparatively less attention devoted to predictions of BCP/solvent/NP systems.^26^ Given recent developments in the dispersion of solid fillers into BCP droplets^27^ it is important to perform a systematic study of the structures formed by systems of BCP/solvent/NPs. The presence of NPs is well known to affect the morphology of BCP-based materials,^19,28,29^ therefore, phase diagrams can guide future experimental explorations of BCP particle morphology in the presence of NPs.

This work aims at first systematically exploring the BCP morphology under confinement due to the presence of a solvent, with emphasis on conditions of geometric frustration. Secondly, we will systematically study the phase behavior of more complex mixtures where NPs are added to BCP particles. To this end, a mesoscopic model will be used called cell dynamic simulation (CDS), which allows for performing a large number of simulations at a modest computational cost.^30^ This is of crucial importance due to the several length scales involved in BCP/solvent/NP hybrid systems: smallest length scales related to the BCP-solvent *ξ* and BCP-NP interface *R*; intermediate ones associated with the BCP mesophase *H*_0_; and the macrophase separation associated with the BCP droplet size *R*_*drop*_. Despite its simplicity, this coarse-grained model has a well-established track record at successfully reproducing the behavior of BCP-based materials including BCP under solvent confinement^31,32^ BCP under external fields^33^ and BCP nanocomposites.^34^ To avoid confusion, we will refer to the BCP-rich droplets as BCP particles or droplets and to the nanoscopic fillers as NPs. In this work we will focus on relatively small NPs, comparable to the BCP-solvent interface *R*∼ *ξ*, smaller than the BCP mesophase *R* ≪ *H*_0_ confined in larger BCP droplets *H*_0_ ≪ *R*_*drop*_.

## Model

We consider a blend of four components: A/B diblock copolymer with degree of polymerization *N*_*A*_ and *N*_*B*_ respectively for the A and B species; a solvent composed of a species C; and a collection of *N*_*p*_ solid NPs with radius *R*. Species A, B and C are characterized by three local volume fractions *ϕ*_*ν*_ with *ν* = A,B,C for the two species of the BCP and the solvent, respectively. Assuming incompressibility, it is possible to specify the state of the system by just two order parameters^35^ defined as *ϕ*(**r**,*t*) = *ϕ*_*C*_ - *ϕ*_*A*_-*ϕ*_*B*_ and *ψ*(**r**,*t*) = *ϕ*_*A*_ - *ϕ*_*B*_, which correspond to the local composition of the solvent and the local composition of the BCP, respectively. The position of NPs is specified by **r**_*i*_ with *i* = 1··· *N*_*p*_, with radius given by *R*.

### Free Energy of the System

The total free energy of the system is given by four components, in units of *k*_*B*_ *T*,

1 $$F=F_{\mathrm{blend}}+F_{\mathrm{pp}}+F_{\mathrm{NP}-\psi }+F_{\mathrm{NP}-\phi }$$

respectively the free energy of the BCP/solvent blend, the particle-particle and the particle-fields interactions.

#### Free Energy of the Blend

The free energy of the BCP/solvent blend can be derived from the classical Ohta-Kawasaki free energy^36^ with contributions from Ito^35^ following the procedure by Avalos et al^31^ leading to

2 $$\begin{array}{cc} F_{\mathrm{blend}}= & \int dx[W\left(\phi ,\psi \right)+\frac{1}{2}D_{\phi }(\nabla \phi {)}^{2}+\frac{1}{2}D_{\psi }\left(\nabla \psi {)}^{2}\right]+\\ & \frac{1}{2}B\int dx\int dx^{\prime }G\left(x,x^{\prime }\right)\left[\psi \left(x\right)-\mathrm{\psi ̅}\right]\left[\psi \left(x^{\prime }\right)-\mathrm{\psi ̅}\right] \end{array}$$

where we define ϕ̅ = ⟨ ϕ­(**x**)⟩ and ψ̅ = ⟨ψ­(**x**)⟩ as the mean values of the two order parameters. The mean value ψ̅ is associated with the asymmetry of the BCP and related to the composition of the chain, *f*_*A*_ = *N*_*A*_/(*N*_*A*_+*N*_*B*_) by ψ̅ = 2*f*_*A*_-1. The interfacial penalty energy for each order parameter is characterized by both *D*_*ϕ*_ and *D*_*ψ*_, which translates into an equilibrium interface thickness $\xi_{\nu }=1/\sqrt{D_{\nu }}$ for each concentration field. The local free energy determines the equilibrium values of both concentration fields in the bulk as the minima of the field

3 $$W\left(\phi ,\psi \right)=\frac{1}{4}(\phi^{2}-1{)}^{2}+\frac{1}{4}(\psi^{2}-1{)}^{2}+\alpha \phi \psi +\beta \phi \psi^{2}$$

where *α* and *β* characterize the coupling between both order parameters. The first two terms are the standard double-well potentials for phase-separating mixtures where equilibrium *ψ*_eq_ = ± 1 and *ϕ*_eq_ = ± 1 can be recovered by minimization of *W*(*ϕ*,*ψ*) in the case *α* = *β* = 0, that is, in the absence of coupling between the two order parameter fields. Parameter *α* introduces the selectivity of the solvent towards positive or negative values of *ψ* while *β* forces the BCP field *ψ* to adopt a homogeneous value within the solvent-rich domains. In particular, a choice *β* > 0 implies that, in order to minimize the term βϕψ^2^, in regions where *ϕ* >0 (solvent-rich) |*ψ*|→ 0, that is, the BCP tends towards its homogeneous, mixed state. On the other hand, in regions where ϕ<0 (BCP-rich) minimization of eq 3 leads to ψ → ±1, that is, demixed state with well-defined A-rich and B-rich regions.

The double integral term in eq 2 is the long range free energy associated with the connectivity of the BCP chain and the parameter *B* is related to the degree of polymerization and the BCP periodicity.^36^ It introduces the Green function of the Laplacian operator ∇^2^ G­(**x**) = −*δ*(**x**).

#### Particle-Particle Free Energy

In order to prevent overlapping and take into account excluded volume interactions, we introduce a pairwise additive contribution to the free energy

4 $$F_{\mathrm{pp}}=\underset{ij}{\sum }V\left(r_{ij}\right)$$

due to a completely repulsive potential which takes the form of a Yukawa-like potential

5 $$V\left(r_{ij}\right)=V_{0}\exp\left[1-r_{ij}/\left(2R\right)\right]/\left[r_{ij}/\left(2R\right)\right]$$

where *V*_0_ sets the strength of the repulsive potential. The cutoff for the potential is set as *V*(*r*>2*R*) = 0.

#### Coupling Free Energy

The interaction between the NPs and the BCP/solvent blend is introduced by coupling the position of the NPs with the order parameters *ψ* and *ϕ* via the two free energies^37,38^

6a $$F_{\mathrm{NP}-\psi }=\sigma_{\psi }\underset{i}{\sum }\int dr\psi_{c}\left(|r-r_{i}|\right){[\psi (r)-\psi_{0}]}^{2}$$

6b $$F_{\mathrm{NP}-\phi }=\sigma_{\phi }\underset{i}{\sum }\int dr\psi_{c}\left(|r-r_{i}|\right){[\phi (r)-\phi_{0}]}^{2}$$

where *σ*_*ψ*_ and *σ*_*ϕ*_ sets the interaction strength between the NP and the field *ψ* and *ϕ*, respectively. Furthermore, parameters *ψ*_0_ and *ϕ*_0_ specify the selectivity of the NPs, as each the free energy is minimized when NPs are segregated within regions of space where *ψ*(**r**) ≈ψ_0_ and *ϕ*(**r**)≈ *ϕ*_0_. Finally, the size and shape of the particle is given by the tagged field^39^

7 $$\psi_{c}\left(r<R\right)=\exp\left[1-\frac{1}{1-(r/R{)}^{2}}\right]$$

and *ψ*_*c*_(*r* ≥ *R*)=0 which has a compact form and vanishing derivative at the cutoff *r*=*R*. This tagged function moves along the center of mass of the particle.

### Dynamics

The dynamics of the two order parameters follow two coupled Cahn–Hilliard^40–42^ equations

8a $$\frac{\partial \psi }{\partial t}=M_{\psi }\nabla^{2}\left(\frac{\delta F}{\delta \psi }\right)$$

8b $$\frac{\partial \phi }{\partial t}=M_{\phi }\nabla^{2}\left(\frac{\delta F}{\delta \phi }\right)$$

where *M*_*ψ*_ and *M*_*ϕ*_ are the mobilities^43^ of the BCP and solvent order parameters *ψ* and *ϕ*, respectively. The dynamics of the system are determined by the functional derivatives of the total free energy of the system, where the two order parameters are coupled by the *α* and *β* terms. Random noise fluxes can be introduced leading to the Cahn–Hilliard-Cook dynamics^44,45^ however, they have been shown to play a secondary role in the formation of BCP structures. For this reason, we assume no thermal fluctuations in the density fields *ψ* and *ϕ*.

The dynamics of the NPs are controlled by the overdamped Langevin equation leading to

9 $$\frac{dr_{i}}{dt}=\frac{f_{i}}{\gamma }+\sqrt{2D}\xi^{i}\left(t\right)$$

where ***ξ*** ^*i*^(*t*) is a random force term that satisfies fluctuation-dissipation theorem

10a $$\left\langle \xi^{i}\left(t\right)\right\rangle =0$$

10b $$\left\langle \xi_{\alpha }^{i}\left(t\right)\xi_{\beta }^{j}\left(t^{\prime }\right)\right\rangle =\delta_{\alpha \beta }\delta_{ij}\delta \left(t-t^{\prime }\right)$$

where $\xi_{\alpha }^{i}$ is the *α* component of the ***ξ*** ^*i*^ vector.

The force acting on particle *i* arises from their interaction with neighboring NPs (via eq 4), and with the medium (both *ψ* and *ϕ* fields, respectively eq 6,7) through

11 $$f_{i}=-\frac{\partial }{\partial r_{i}}\left(F_{\mathrm{pp}}+F_{\mathrm{NP}-\psi }+F_{\mathrm{NP}-\phi }\right)$$

On the other hand, the coupling free energy terms 6a and 6b affect the *ψ* and *ϕ* fields as chemical potential terms, respectively, *δ* *F*_*NP*‑*ψ*_/*δψ* and *δ* *F*_*NP*‑*ϕ*_/*δϕ* on the Cahn–Hilliard equations shown in eq 9,10.

### Observables

#### Radially Aligned Nematic Order Parameter

In order to capture the symmetry associated with the onion-like morphology (see Figure 2e) , we define a radially-aligned nematic-like order parameter

12 $$S_{r}=2\langle \mathrm{n̂}\cdot \mathrm{r̂}\rangle^{2}-1$$

where **n̂** = ∇ψ/|∇ψ| is the normalized gradient of the order parameter *ψ* while **r̂** is the radial unit vector from the center of mass of the droplet (see the center of the concentric layers in Figure 2c).

**Figure 1:**
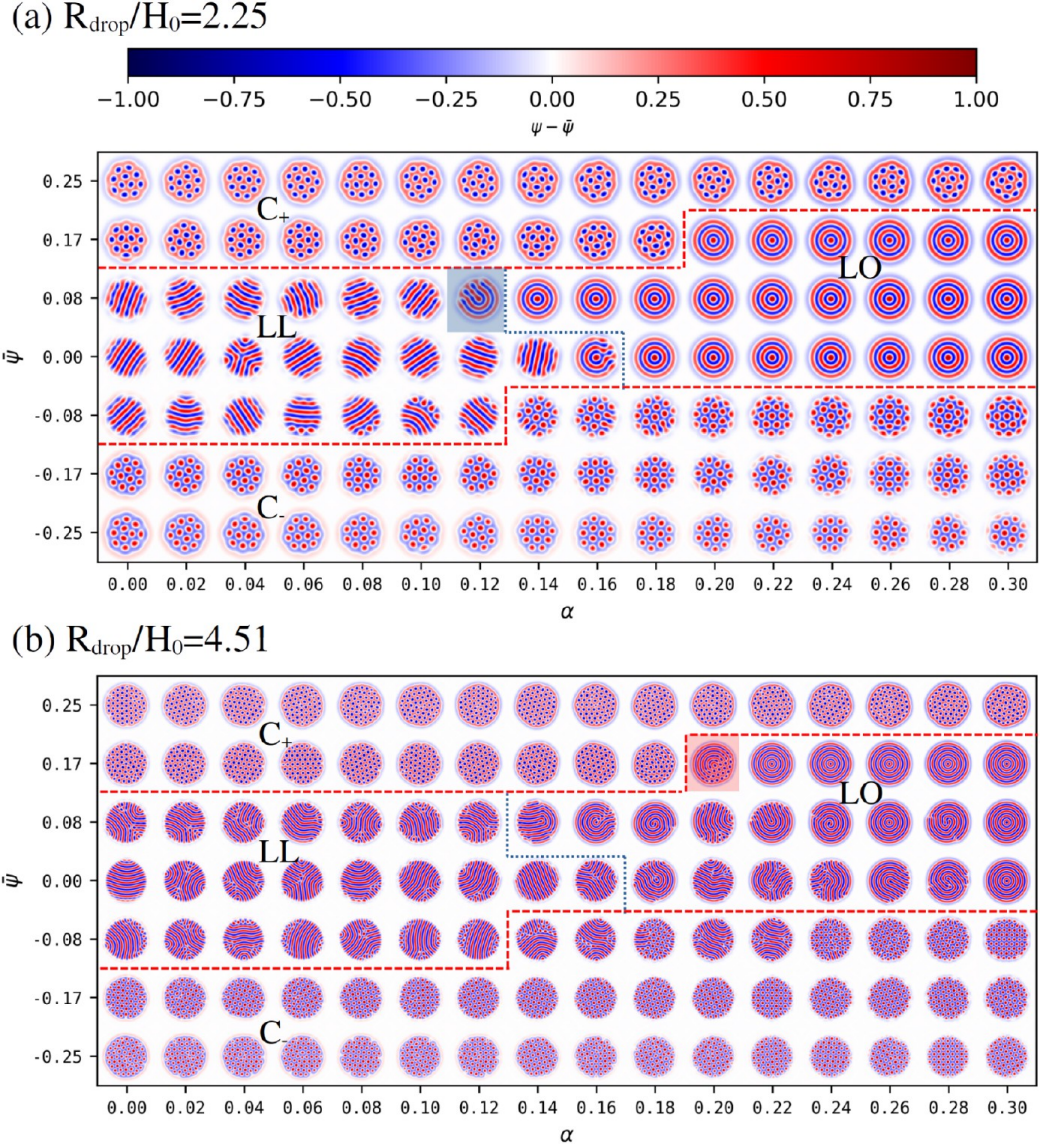
Phase diagram of the BCP/solvent system in terms of the BCP composition ψ̅ and the selectivity *α*. For each point (*α*,ψ̅), a snapshot of the system is shown as the colormap of the field *ψ*-ψ̅. Phase boundary lines are superimposed to guide the eye, shown as red dashed lines to separate circular-lamellar morphology, and blue dotted line to separate onion-ellipsoid ones. The BCP/solvent fraction is kept constant at ϕ̅ = 0.5 while the system size is explored as (a) *L*_*x*_ = *L*_*y*_ = 64 and (b) *L*_*x*_ = *L*_*y*_ = 128. In terms of BCP periodicity: *R*_*drop*_/*H*_0_ = 2.25 and 4.51 respectively for (a) and (b). See Fig. S2 for a larger *R*_*drop*_/*H*_0_ = 9.0 case.

**Figure 2:**
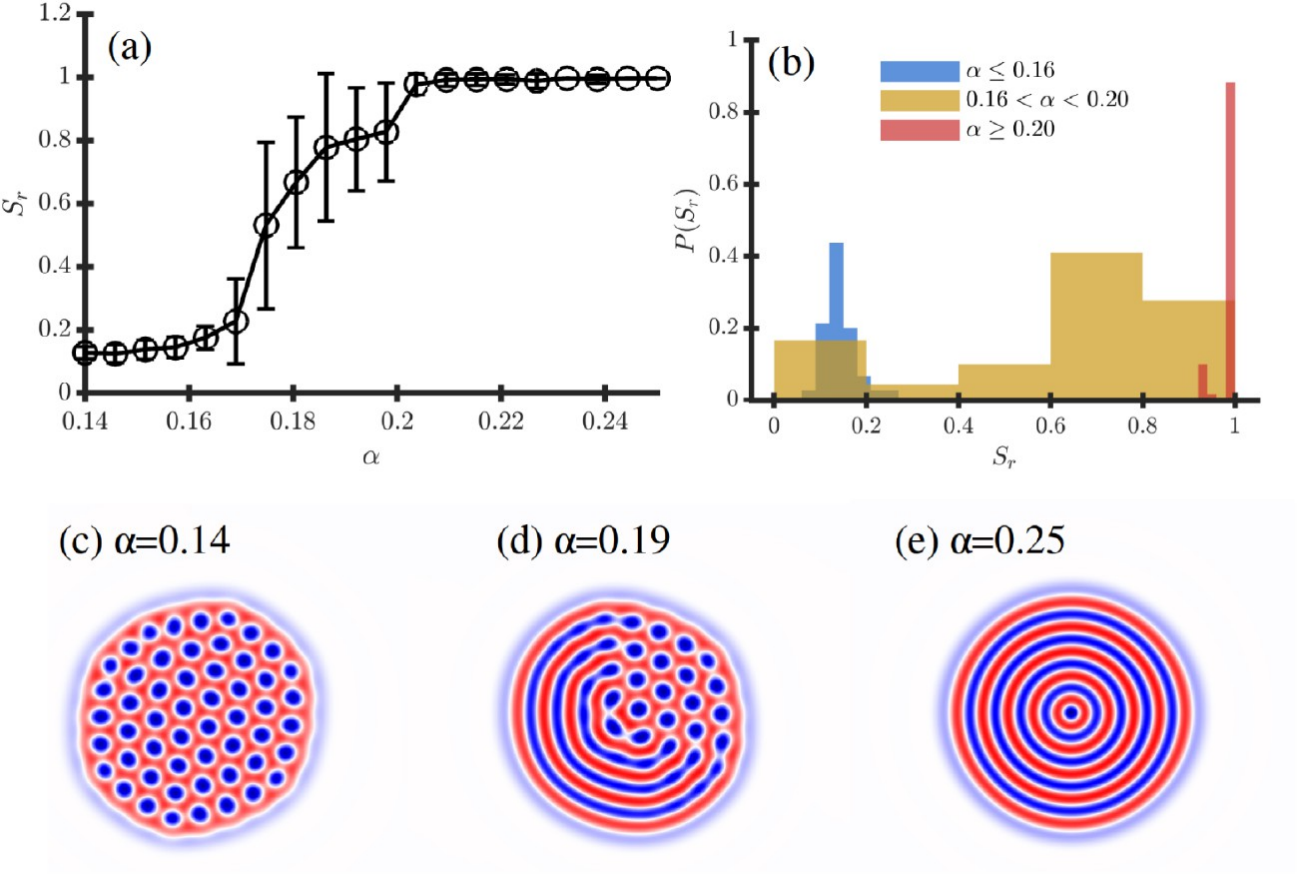
Mixed Janus-like morphology for ψ̅ = 0.16 in terms of *α* characterized in (a) by _S*r*_ and its histogram *P*(*S*_*r*_) in (b). Representative snapshots are shown in (c) *α* = 0.14, (d) 0.19 and (e) 0.25.

**Figure 3:**
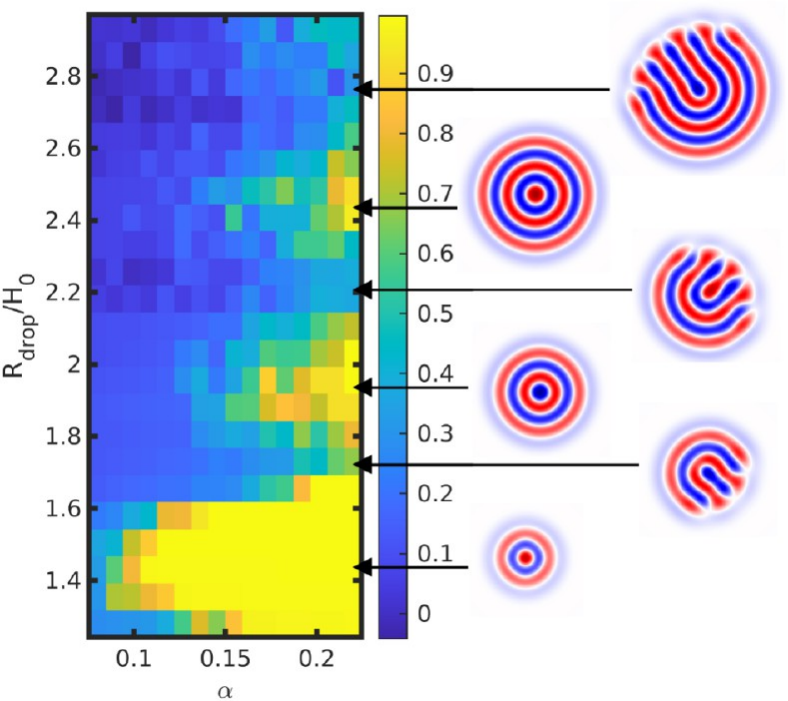
Frustration in symmetric BCP droplets with ψ̅ = 0, quantified by *S*_*r*_ in terms of droplet size *R*_*drop*_ and solvent selectivity *α*. Representative snapshots are shown on the right with each arrow pointing to the corresponding simulation. Each *S*_*r*_ is an average over 10 independent runs.

**Figure 4:**
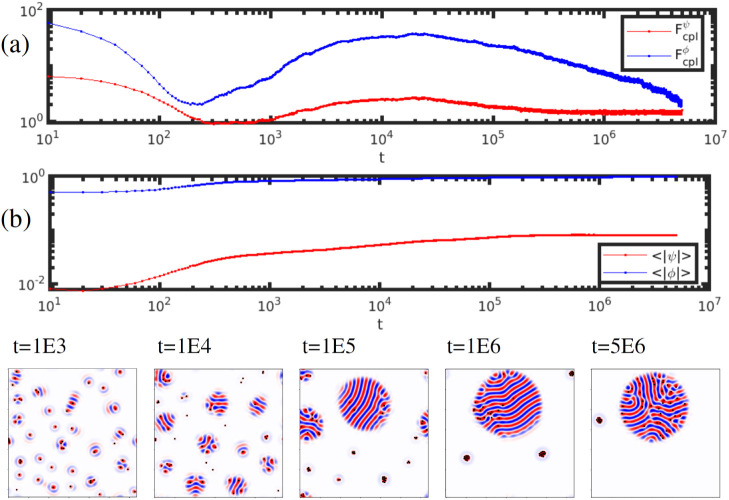
NP distribution over time: (a) shows the time evolution of the two particle-field contributions to the free energy (see eq 6). (b) shows the time evolution of the average absolute value of the two order parameter fields ⟨ |*ψ*| ⟩ and ⟨ |*ϕ*| ⟩ . Representative snapshots over time (see top values for simulation times) are shown at the bottom, where NPs are shown as solid black circles.

#### Hexatic Order Parameter

The six-fold hexatic phase of the BCP is characterized by a standard order parameter given a collection of coordinates **r**_i_

$$\Psi_{6}=\left\langle \frac{1}{N_{i}}\left|\overset{N_{i}}{\underset{j}{\sum }}e^{6i\theta_{ij}}\right|\right\rangle$$ 13

where *θ*_*ij*_ is the angle sustained by the vector between particle *i* and *j* center of mass, with particle *i* having *N*_*i*_ neighbors. Specifically, we will apply this order parameter to the center of mass of each BCP domain, which is obtained from standard cluster analysis tools. We can differentiate between the *positive* and *negative* domains of the BCP (see, for instance, Figure 1a for *α* = 0 and ψ̅ ∼ ± 0.25 to see red and blue domains). This allows to define the positive/negative order parameter $\Psi_{6}^{+,-}$ for each distinct set of coordinates.

### Simulation Details

A standard CDS approach is used to discretize the two coupled time-dependent Ginzburg–Landau equations -eq 9,10 with a discrete grid size *δ* *x* and time step *δ* *t*. Meanwhile, a standard forward Euler scheme is used to discretize the Brownian dynamic eq 11 with the same time step *δt*. A more detailed description of the overall discretization scheme can be found in a recent review.^46^ We select *δx* = 1.0 and *δt* = 0.1. Subsequently, we will express lengths in number of grid points and times in number of time steps.

We use standard CDS parameters *D*_*ψ*_ = *D*_*ϕ*_ = 1.0 and *B* = 0.2, which leads to BCP periodicity *H*_0_≈ 8 grid points. The mobilities are set to *M*_*ψ*_ = *M*_*ϕ*_ = 0.25 for simplicity. The choice of parameters can be roughly mapped to a BCP melt with Flory-Huggins parameter *χ* *N* ∼ 12,^36^ indicating a weakly segregated BCP melt. NPs are simulated with standard hybrid CDS parameters^46^ *R* = 1.25, *σ*_*ψ*_ = *σ*_*ϕ*_ = 1, *D* = 0.05 and *γ* = 2.3.

At *t* = 0 systems can be initialized from a random configuration where both *ψ* and *ϕ* are assigned random values around their average ψ̅ and ϕ̅ respectively, with amplitude 0.1. The position of the NPs **r**_*i*_ can equally assigned randomly at *t* = 0. This initial configuration will be used for cases when the focus of the study is the kinetics of the system. Contrary to that, for cases where the focus is on the steady-state morphological behavior of the BCP droplet, we prescribe the droplet morphology at *t* = 0 by defining a droplet with size *R*_*drop*_ leading to *ϕ*(*r*<*R*_*drop*_) = −1 and *ϕ*(*r*>*R*_*drop*_) = +1 where *r* is the distance to the center of the system box. The BCP order parameter is randomly initialized with amplitude 0.1 while NPs are randomly located within the prescribed droplet.

## Results

In this section we will firstly revisit the phase behavior of BCP/solvent blends by systematically studying the morphology of a BCP droplet under different conditions of solvent selectivity, BCP composition and droplet size, where we will address the role of geometric frustration due to confinement. Secondly, we will study the impact of NPs, which will considerably change the droplet morphology.

In this work, we will explore *α* ≥ 0, *i.e.*, the solvent will either be selective toward the A monomers (*ψ* >0) or neutral (*ψ* = 0). The results for A-selective solvents can be trivially extended for B-selective solvents by considering negative *α* values.

### Block Copolymer/Solvent Blend

In order to establish the phase behavior of BCP droplets in solvents, we first study the morphology of a BCP droplet with size *R*_*drop*_ in a solvent with selectivity *α*. Figure 1 shows the morphological phase behavior of a BCP/solvent blend for two droplet sizes *R*_*drop*_/*H*_0_ = 2.25 and *R*_*drop*_/*H*_0_ = 4.51 (a) and (b), respectively, exploring the system size with (a) *L*_*x*_ = *L*_*y*_ = 64 and (b) *L*_*x*_ = *L*_*y*_ = 128. A larger system size has been explored in Fig. S2 where we observe the same qualitative behavior as Figure 1b. Simulations are initialized with a prescribed droplet morphology, observing the same qualitative behavior as a randomly-initialized run, as shown in Fig. S1 for *R*_*drop*_/*H*_0_ = 2.25. In each case, the solvent selectivity *α* and the BCP composition ψ̅ are explored systematically. For each simulation we provide a snapshot of the resulting BCP morphology after *N*_steps_ = 10^6^ time steps.

Three different morphologies can be roughly distinguished, which in Figure 1 are separated by red dotted lines for the circular-lamellar boundary and blue dotted for the onion-ellipsoid one:

1. Approximately symmetric BCP with ψ̅ ∼ 0 can display a layered lamellar (*LL*) morphology for small values of *α* ≤ 0.15. The lack of a majority/minority species in the BCP leads to a stable lamellar morphology characterized by alternating A/B domains. Furthermore, the solvent is approximately nonselective leading to perpendicular lamellar domains with respect to the BCP/solvent interface. Importantly, the BCP droplet becomes elongated in order to maximize the alternating lamellar morphology. As this is a consequence of frustration, elongation is much more profound for behavior for smaller droplets in (a) rather than larger ones in (b). Elongated striped lamellar morphologies has been widely found in experiments^47^ and studied in simulations and theory.^48^

2. As the solvent becomes selective for *α* ≥ 0.15 while ψ̅ ∼ 0, the BCP acquires an onion-like lamellar (*LO*) morphology reminiscent of multilayered vesicles. Since we are only exploring positive *α* > 0 selectivity, we observe stable onion morphology with A (positive) domains exposed into the solvent. One may notice that the onion morphology is rarely defect-free as the droplet sizes are larger in (b), but can form defect-free equilibrium morphologies in (a), as surface effects are dominating over bulk ones. Such onion-like layered structures are commonly found in experiments^49^ and theory.^31^

3. In Figure 1, for highly asymmetric BCP compositions ψ̅ = ± 0.25, hexagonal circular domains are stable (*C*). This are characterized by an hexagonal lattice of positive or negative circular domains in a negative or positive matrix (C±). Again, the value of *α* dictates the wetting properties of the BCP and therefore the monomer which is exposed into the solvent, forming a layer.

The phase diagram in Figure 1a can be compared with Fig. S1 for the same parameters but a completely random initial condition after *N*_*steps*_ = 10^7^ steps: The phase behavior is consistent regardless of the initial condition, which supports the use of a prescribed droplet as the initial condition for computational efficiency.

In Figure 1a, the transition from the layered lamellar to the onion lamellar one is marked by an intermediate frustrated phase as shown, for instance, for *α* = 0.12 and ψ̅ = 0.08 (see blue shaded square), in accordance to previous simulation^32^ and experimental results (see Figure 5 in Ref.^19^ However, for larger BCP droplet sizes in (b) we can also observe a different intermediate morphology that connects the hexagonal morphology with the onion one, for instance, for *α* = 0.2 and ψ̅ = 0.17 (see red shaded square). This morphology is marked by a segregation of the morphology of the BCP into two distinct regions: one region of the droplet is onion-like and the other one is hexagonal, in a Janus-like type of self-assembly. Fig. S3 shows a more focused phase exploration in the transition region where both the circular and onion phase can be observed, as well as a several Janus-like particles with various proportions of the two equilibrium phases.

**Figure 5:**
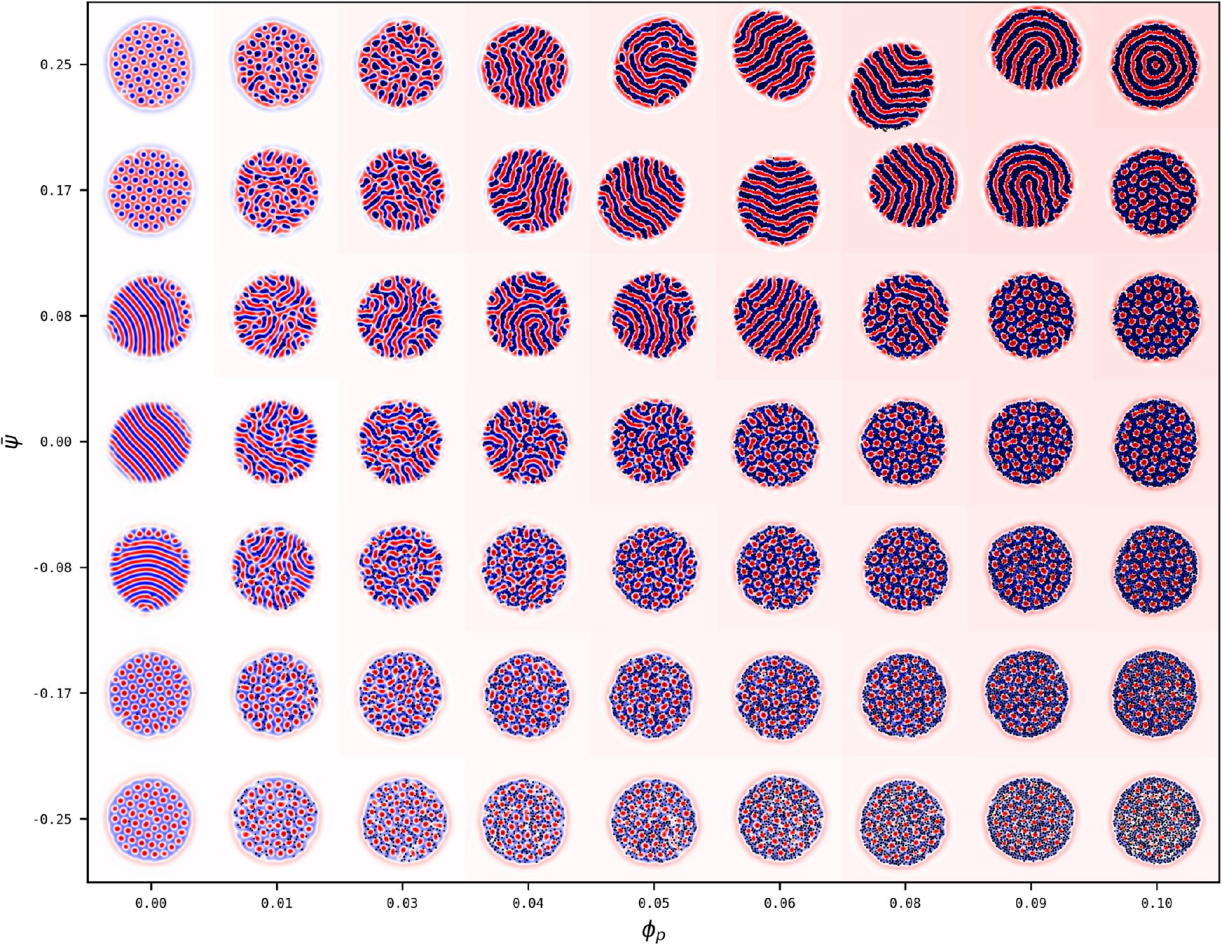
Phase diagram of BCP droplets with composition ψ̅ in a neutral solvent with *α* = 0.0 in the presence of a concentration *ϕ*_*p*_ and NP affinity *ψ*_0_ = −0.5. The shift in background color (see color bar) is due to the addition of NPs that disturb the overall value of *ψ*-ψ̅ and modifies the value of *ψ* in the solvent bulk. The same color bar as in Fig. 1 is used.

In order to quantify the transition between the hexagonal and the onion phase and characterize the its prevalence, we will use the radial order parameter *S*_*r*_ (see eq 15) which takes value *S*_*r*_ ∼ 0 for the hexagonal phase and *S*_*r*_ ∼ 1 for an onion morphology with radial symmetry. Figure 2 shows the transition from hexagonal to onion in more detail by calculating the radial order parameter *S*_*r*_ in terms of the selectivity *α* for ψ̅ = 0.16 for a droplet with size *R*_*drop*_/*H*_0_ = 4.51. The error bars are calculated from 20 independent realizations for each value of *α*. One may notice large variability in the region 0.16< *α* <0.20, which may suggest the coexistence of different morphologies leading to different *S*_*r*_ values: circular morphology *S*_*r*_ ∼ 0, onions with *S*_*r*_ ∼ 1 and this Janus-like morphology leading to intermediate 0 ≲ *S*_*r*_ ≲ 1 values. The probability distribution of *S*_*r*_ values indicates that there is no clear bimodal distribution of *S*_*r*_ values in the coexistence region, instead, intermediate *S*_*r*_ are present in the system.

Figures 1 and 2 suggests that the kinetic pathway connecting the hexatic and the onion phase is achieved via a steady-state Janus-like morphology for asymmetric BCP droplets. Experiments by Hawker *et al* ^19^ have shown the coexistence of onion-like and stacked lamellar morphology in a Janus-like fashion (see Figure 5 in that work).

Furthermore, experiments by Yabu et al.^50^ have shown cross-sectional TEM images (see Figure 4e in that work) of onion-like and spherical morphology with spatial segregation across the droplet. To the best of our knowledge we report the first simulations on hexatic/onion coexistence in BCP particles. This is in accordance to the well-reported kinetic pathway of symmetric BCP droplets during the transition from ellipsoid to onion morphology. Future work may be devoted to study the stability of the Janus-like morphology.

In Figure 1 we have considered only two droplet sizes *R*_*drop*_/*H*_0_ = 2.25 and *R*_*drop*_/*H*_0_ = 4.51 (in addition in Fig.S2 we show *R*_*drop*_/*H*_0_ = 9.0), while geometric confinement is know to play a crucial role in the degree of frustration and the resulting morphology.^31,51^ This suggests that a systematic exploration of droplet size may allow to gain insight over the role of geometric frustration. Intermediate defect-full morphologies can be observed in Figure 1a for ψ̅ = 0.08 and *α* = 0.12. Figure 3 shows a systematic exploration of the radial order parameter *S*_*r*_ in terms of *R*_*drop*_/*H*_0_ and *α*, in order to better understand the role of frustration in the self-assembled structures. We note that we keep *B* (and thus *H*_0_) constant and explore *R*_*drop*_, in contrast with Ref.^31^ As expected, the defect-free onion morphology occurs for certain values of *R*_*drop*_/*H*_0_ where the droplet size is commensurable to the periodicity of the lamellar. When this is not the case, frustrated intermediate structures can be observed. Interestingly, the repeating vertical pattern where *S*_r_ ∼ 1 observed in Figure 3 is reminiscent of the perforated lamellar morphology vs parallel lamellar found in thin films.^51^

### Block Copolymer/Solvent/Nanoparticles

Once the phase behavior of a BCP/solvent blend has been systematically established, the effect of a concentration *ϕ*_*p*_ of NPs can be studied under various conditions of NP affinity, solvent selectivity and BCP composition. In this section we will first study the NP distribution in the dilute regime *ϕ*_*p*_→ 0, then consider the system phase behavior under generic NP conditions and finally focus on highly frustrated phases.

#### NP Distribution in BCP Droplets

Figure 4 shows the NP assembly within the BCP/solvent blend over time. We study the implications of the two coupling terms eq 6,7 by considering selective NPs with respect to the solvent *ϕ*_0_ = −1 and the BCP melt *ψ*_0_ = +0.5. This implies that NPs are completely miscible within phase A of the BCP. We do not choose a *ψ*_0_ = 1.0 value because the BCP melt does not reach completely |*ψ*|=1 values in bulk, due to the weak segregation conditions for *B* = 0.2. A simple instance of symmetric BCP ψ̅ = 0 in a neutral solvent *α* = 0 is chosen. In equilibrium, we expect all NPs to be dispersed within the solvent-poor (BCP-rich) regions of the system with *ϕ* ∼ −1 in order to minimize the particle-solvent interaction eq 7. Similarly, in order to minimize the particle-BCP interaction eq 6, NPs are expected to segregate within the *ψ* ∼ +1 domains in the BCP.

In Figure 4, initializing from a homogeneous disordered state- for the *ϕ* and *ψ* fields and the NP coordinates **r**_*i*_- we can study the coarsening of the blend and, simultaneously, the NP distribution. For short times *t* ∼ 10^3^ we can observe that NPs are nucleating the formation of positive *ψ* > 0 domains of the BCP due to the particle-BCP interaction. For intermediate stages of phase separation , *t* ∼ 10^4^, small droplets with few lamellar periods are formed. Since the droplets are small, they can easily acquire the characteristic stripe ellipsoid morphology, with NPs segregated within the positive domains of the BCP in order to minimize $F_{cpl}^{\psi }$ . As droplets grow via diffusion from small to large droplets, NPs populate the positive domains, while some may remain temporally within the solvent phase due to the evaporation of the hosting droplet. For late times these NP clusters -see snapshots for *t* = 10^5^ and *t* = 10^6^- eventually coalesce into the largest droplet as shown for *t* = 5× 10^6^.

The snapshots in Figure 4 show that there are various time scales at play dictating the time evolution of the system: there is a fast droplet nucleation which can be seen as rapid changes in ⟨|*ψ*|⟩ and ⟨|*ϕ*|⟩ in (b), indicating that BCP-rich droplets are formed, and in-droplet mesophase separation takes place (see red/blue domains formed within droplets for *t* = 10^3^). A slower time scale is associated with the Ostwald ripening as droplets grow in time and shown as a slow grow in both ⟨|*ψ*|⟩ and ⟨|*ϕ*|⟩. Finally, the slowest time scale is associated with the coalescence of NP-rich droplets that eventually merge with the largest droplet, characterized by a slow decrease in the coupling free energies $F_{cpl}^{\psi }$ and $F_{cpl}^{\phi }$ in (a), as shown in eq 6. The presence of these various slow time scales indicates that, in order to be able to reasonably explore the phase behavior of BCP droplets in the presence of NPs, we will consider a prescribed initial configuration at *t* = 0, which assumes that (1) the solvent has been removed from an initial droplet and (2) all NPs are initially within such a droplet. This allows to overcome the slow time scales and focus on the dynamics associated with the development of the internal structure of the BCP in the presence of NPs.

#### Phase Behavior

In this section we systematically study the morphology of a BCP droplet in the presence of a concentration *ϕ*_*p*_ of NPs. We consider several limiting cases of NP affinity *ψ*_0_ and solvent selectivity *α*.

We first consider NPs with affinity toward negative domains *ψ*_0_ = −0.5 in a neutral solvent with respect to the BCP *α* = 0.0. In Figure 5, in the absence of NPs, *ϕ*_*p*_→ 0, we recover the three expected BCP droplet morphologies: hexatic positive domains for ψ̅ < −0.1, lamellar domains with −0.1 < ψ̅ < 0.1 and hexatic negative domains for ψ̅ > 0.1. The presence of NPs can substantially deform the BCP droplet morphology. For ψ̅∼ 0 NPs can modify the overall effective composition of the BCP droplet, as NPs segregate and swell the negative BCP domains. This leads to a lamellar-to-hexatic transition which can be observed as a progressive shortening of the elongated lamellar domains. On the other hand, when NPs segregate within the majority domains ψ̅ < −0.1, the NPs do not significantly modify the BCP morphology as the effective concentration of the NPs over the BCP droplets is only moderate: NPs shrink the size of the positive circular BCP domains -due to the swelling of the negative ones- and significantly reduce the degree of hexatic order, which we can attributed to the considerable thermal fluctuations introduced by the NPs. Both the circle-to-lamellar (ψ̅ ∼ 0.2) and the lamellar-to-circle (ψ̅ ∼ 0) transitions are due to the effective changes in the BCP composition due to the presence of selective NPs. This type of transition has been widely reported in the literature of BCP nanocomposites and BCP/homopolymer blends, both in experiments^52–54^ and simulations.^28,55–57^ The red shift in the background color is due to the changes in the overall ψ̅ following the addition of NPs. For pure BCP/solvent blends the boundaries between different morphologies are relatively sharp, as shown in Figure 1- especially (a) for smaller droplets- which allowed to clearly identify phase changes and establish dotted lines marking transition regions. The presence of NPs, instead, greatly disturbs the BCP morphology and defects are more prevalent than in the pure BCP case. For this reason we will resort to order parameters to quantify morphological changes instead of visual inspection to draw boundary lines.

At the top of the phase diagram in Figure 5 there is a hexatic-to-lamellar transition as the NPs swell the hosting negative (minority domains) as shown in Figure 6a where the hexatic order parameter $\Psi_{6}^{-}$ decreases rapidly. Importantly, there is a secondary transition where an emergent onion-like morphology is formed for high NP concentration with *ϕ*_*p*_ ∼ 0.1. This onion morphology is not present in the absence of NPs, as the solvent is nonselective therefore favoring stacked lamellar morphology. This is due to the role of NPs at effectively changing the overall solubility of the droplet: NPs are strongly insolvable in the solvent, therefore, a protective layer of A monomers is preferred in order to minimize the exposure of NPs to the solvent. This appears to be enough to stabilize the onion morphology.

**Figure 6:**
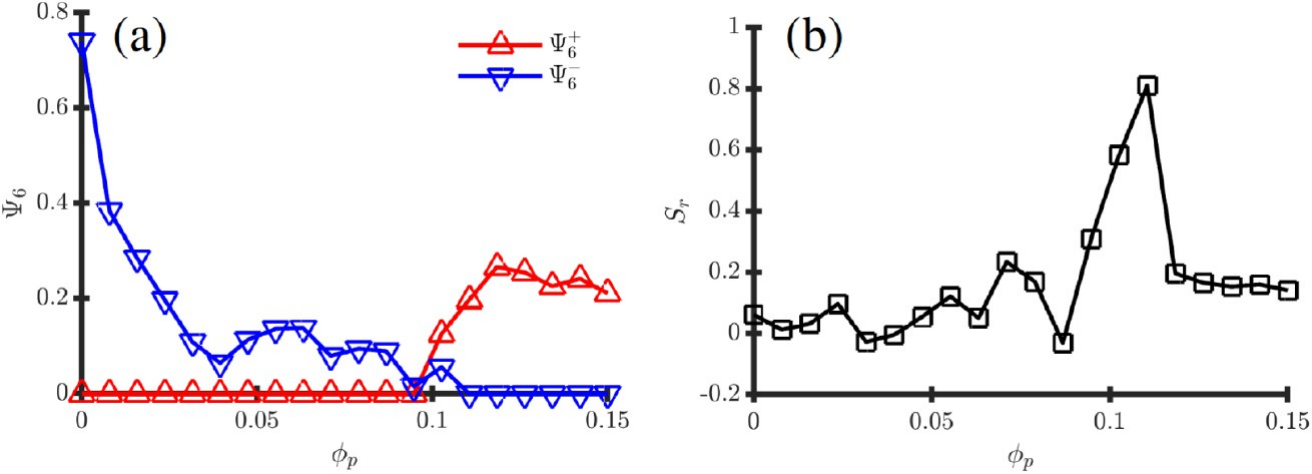
Observables curves associated with the phase transition depicted in Fig.[Fig fig5] with fixed ψ̅ = 0.25. (a) shows the hexatic order parameter Ψ_6_ calculated both for positive (red) and negative BCP domains (blue) in function of *ϕ*_*p*_; (b) shows the radial order parameter *S*_*r*_.

This sphere-to-ellipsoid-to-onion transition is quantitatively studied in Figure 6 by calculating observables that quantify the BCP morphology as NPs are added into the BCP droplet. Figure 6a shows that the original hexatic order $\Psi_{6}^{-}$ decreases rapidly as a small concentration of NPs are added, due to the large fluctuations that NPs introduce in the lattice. For *ϕ*_*p*_ ≳ 0.08, Figure 6b shows the emergence of radial lamellar order with *S*_*r*_ ∼ 0.9, indicating the emergence of onion-like morphology due to the effective solubility changes in the droplet. Exploring higher values of *ϕ*_*p*_ beyond 0.1 leads to another transition where radial order decreases *S*_*r*_ ∼ 0.2, giving way to an inverted hexatic order characterized by $\Psi_{6}^{+}∼0.25$ . One can easily notice that this is simply a consequence of the large concentration of NPs rendering the yellow phase effectively the minority one and thus forming an hexatic morphology. This is easily seen in Figure 5, for instance, for ψ̅ = 0 and *ϕ*_*p*_ > 0.05 where the same mechanism takes place.

Figure 7 shows the phase diagram of a surface fraction *ϕ*_*p*_ of negatively selective NPs with *ψ*_0_ = −0.5 in a BCP droplet immersed in a solvent with selectivity *α* = 0.3 toward the A monomers of the BCP. For *ϕ*_*p*_ → 0, the BCP notably acquires an onion morphology for symmetric BCP ψ̅∼ 0 and hexatic circles otherwise. Note that since the solvent is A-selective for ψ̅ = 0.25, when the BCP droplet is A-majority (continuous) B-minority (isolated circular domains), there is a layer of A monomers exposed into the solvent. For ψ̅ ∼ 0.25, adding NPs can trigger a circle-to-lamellar transition due to the effective swelling of NPs that segregate within the circular domains.

**Figure 7:**
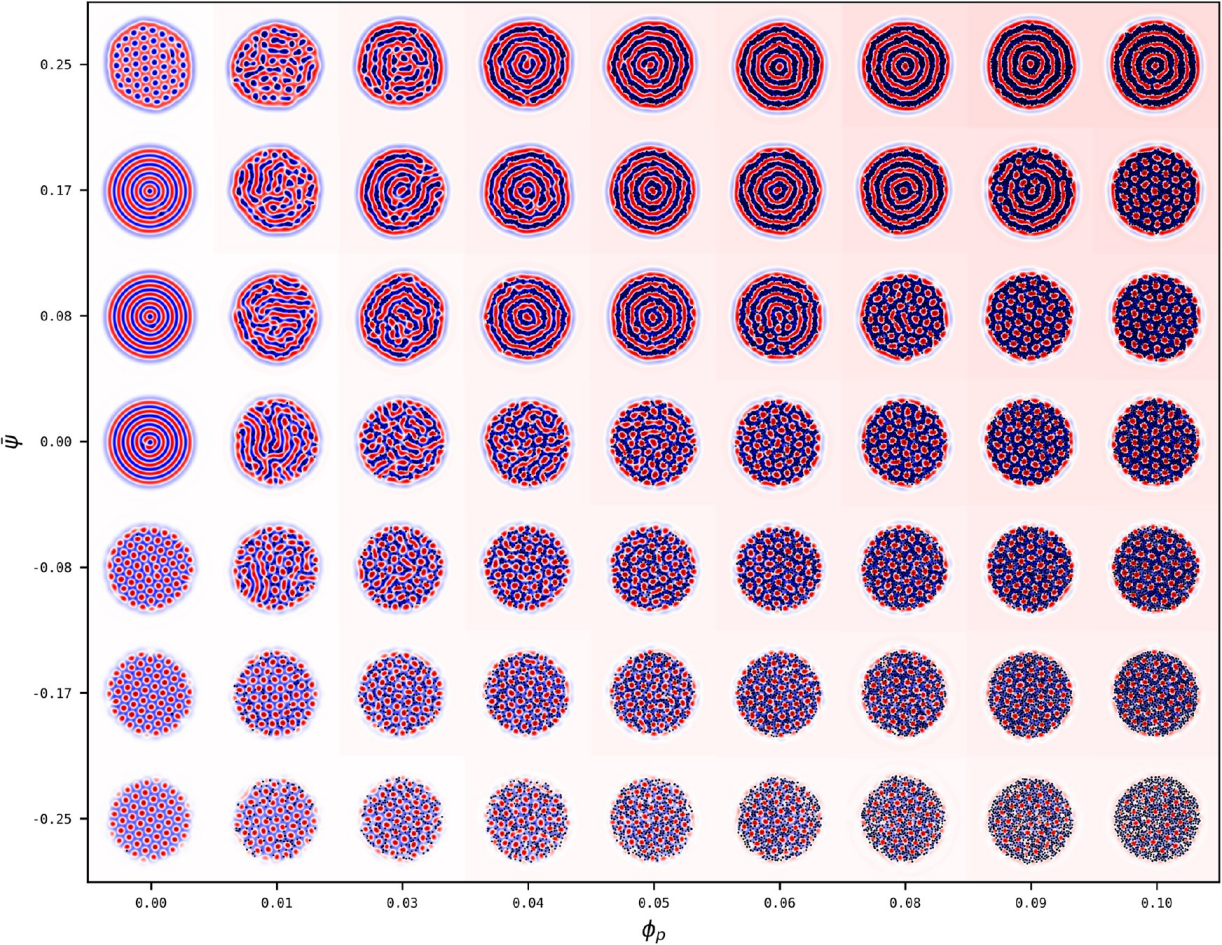
Phase diagram of BCP droplets with composition ψ̅ in a selective solvent with *α* = 0.3 in the presence of a concentration *ϕ*_*p*_ and NP affinity *ψ*_0_ = −0.5.

Figure 8 quantifies the hexatic-to-onion morphology for a representative value ψ̅ = 0.25 of the BCP composition, via the relevant order parameters. The dissolution of the hexatic order can be tracked in (a) where it can be seen to quickly be reduced in the presence of NPs. The alignment of the lamella is onion-like due to the combined effect of the solvent, that promotes a layer of A-monomers exposed into the solvent, and the NPs, which are solvophobic (*ϕ*_0_ = −1) and also prefer segregating into the negative layer of the BCP (*ψ*_0_ = −0.5). The onion-like morphology is robust upon the addition of NPs, as quantified in Figure 8b. On the other hand, when the BCP is ψ̅ ≤ 0.17, NPs promote a circular morphology, which is most prevalent across the phase diagram.

**Figure 8:**
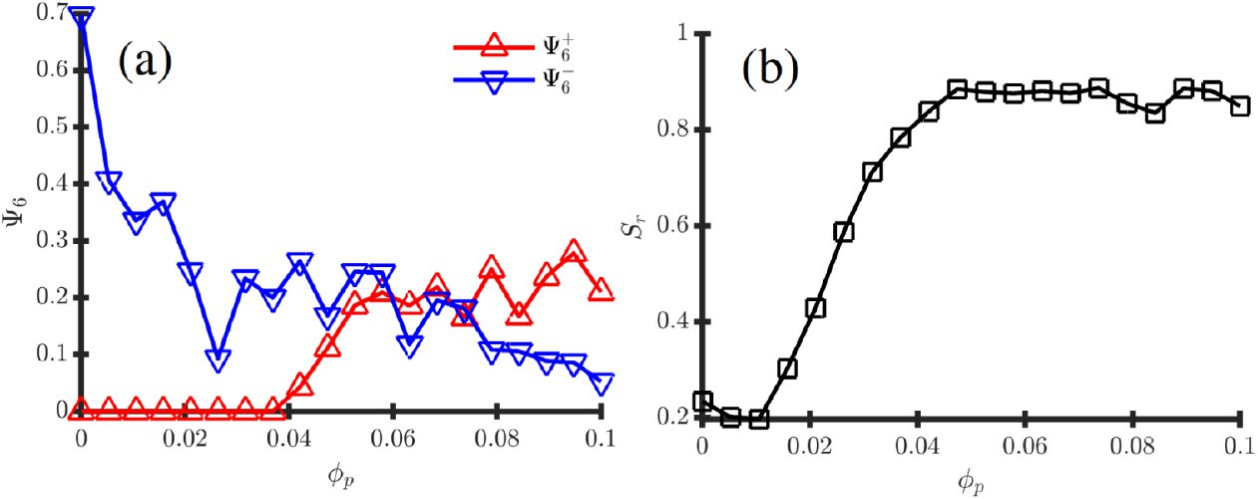
Observables curves associated with the phase transition depicted in Fig.[Fig fig7] for fixed ψ̅ = 0.25. (a) shows the hexatic order parameter Ψ_6_ calculated both for positive (red) and negative BCP domains (blue) in function of *ϕ*_*p*_; (b) shows the radial order parameter *S*_*r*_.

Contrary to the *α* = 0 case in Figure 6, the morphological curves in Figure 8 follow a more monotonic behavior, agreeing with the visual inspection in Figure 7. In 8 (a) the hexatic order decreases slowly as NPs are added into the system. Meanwhile, the onion morphology emerges progressively with *ϕ*_*p*_ until *S*_*r*_ ∼ 0.95 in Figure 8b, indicating that the NP-induced onion morphology is stable over a wide range of NP concentrations, contrary to the mechanism described in the *α* = 0 case.

A complementary scenario of Figure 5 (where NPs are B-selective in a neutral solvent) and Figure 7 (where NPs are B-selective in an A-selective solvent) is given in Figure 9 where NPs are A-selective with *ψ*_0_ = +0.5 in a A-selective solvent with *α* = 0.3. We note that the missing case, *α* = 0.0 and *ψ*_0_ = +0.5 is trivially obtained from Figure 5 by inverting the *Y* axis. The key difference between Figures 7 and 9 is that A-selective NPs can segregate into the wetting layer of A-monomers that form the BCP-solvent interface. This leads to a prevalence of intermediate layered morphologies for moderate values of *ϕ*_*p*_ and less frequent onion morphologies.

**Figure 9:**
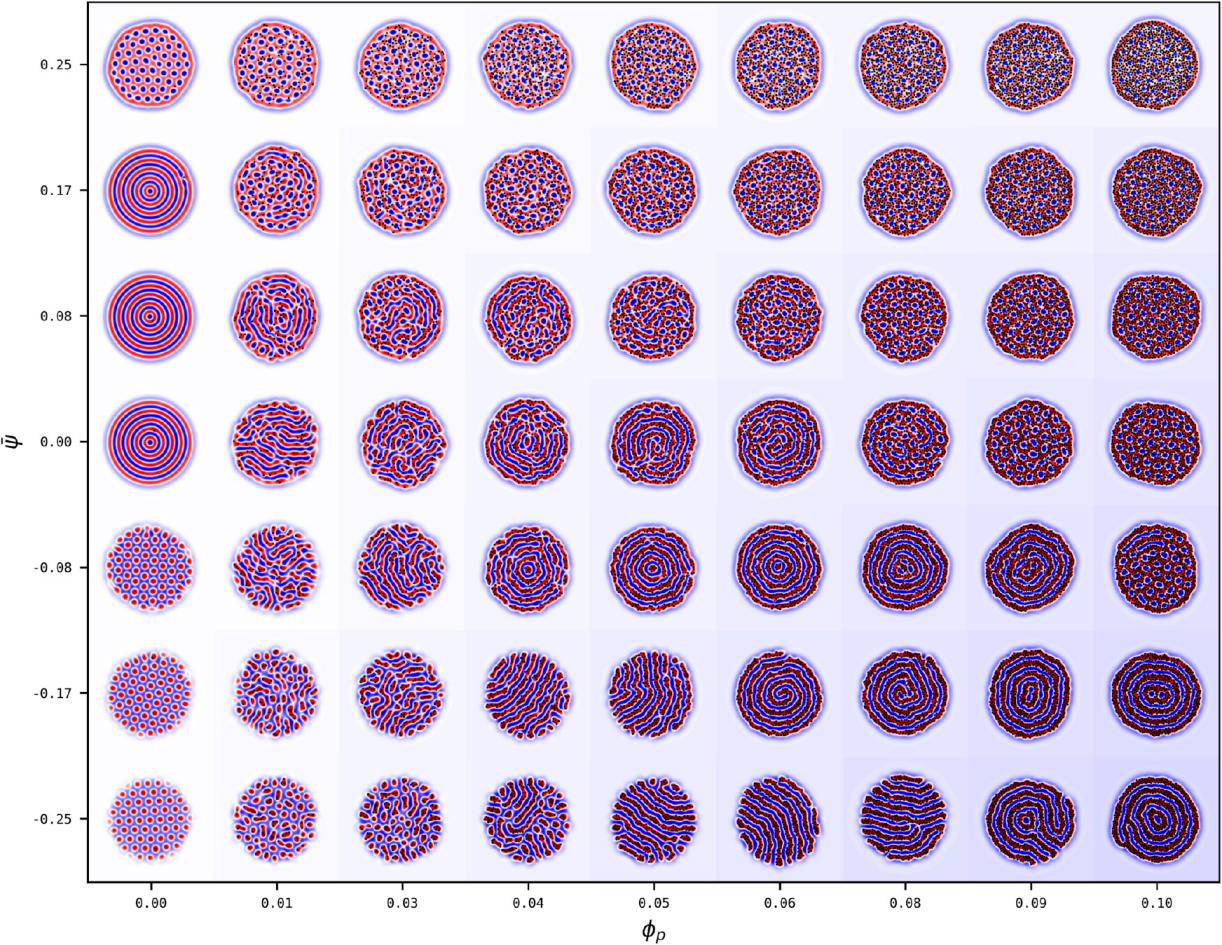
Phase diagram of BCP droplets with composition ψ̅ in a selective solvent with *α* = 0.3 in the presence of a concentration *ϕ*_*p*_ and NP affinity *ψ*_0_ = +0.5.

On top of the pure BCP droplet forming an onion morphology in Figure 1, several instances of onion-like morphology have been found in Figure 5 for NPs in a neutral solvent for the BCP, and Figures 7 and 9 for NPs in a selective solvent. Despite their similar structure, a closer look can distinguish each case: positive-selective NPs in a selective solvent segregate toward the BCP-solvent interface as shown in Figure 9 for ψ̅ = −0.25 and *ϕ*_*p*_ = 0.1; negative-selective NPs in a selective solvent in Figure 7 for ψ̅ = 0.25 and *ϕ*_*p*_ are segregated within the second outer layer, protected from the solvent by the BCP-solvent interface. Similarly, in a neutral solvent in Figure 5 for ψ̅ = 0.25 and *ϕ*_*p*_, NPs are not exposed into the solvent.

**Figure 10:**
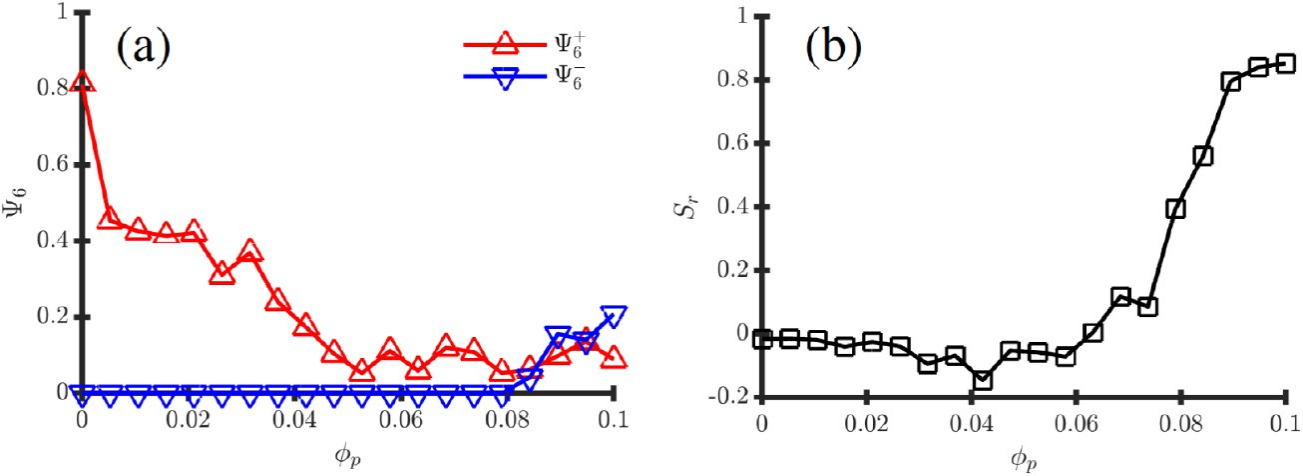
Observables curves associated with the phase transition depicted in Fig.[Fig fig9] with *ψ*_0_ = 0.5, *α* = 0.3 and ψ̅ = −0.25.(a) shows the hexatic order parameter Ψ_6_ calculated both for positive (red) and negative BCP domains (blue) in function of *ϕ*_*p*_; (b) shows the radial order parameter *S*_*r*_.

Figure 11 compares the kinetic pathway toward the NP-induced morphology along with the neat BCP onion phase for *ϕ*_*p*_ = 0, *α* = 0.3 and ψ̅ = 0. As expected, the pure BCP phase reaches a steady state onion morphology the fastest, compared to the two selective solvent *α* = 0.3 cases (red squares for ψ̅ = 0.25, *ϕ*_*p*_ = 0.1 and *ψ*_0_ = −0.5 and green triangles with ψ̅ = −0.25, *ϕ*_*p*_ = 0.1 and *ψ*_0_ = 0.5). This difference in time scales suggests that there is a slower dynamics associated with the degrees of freedom provided by the NPs, as NPs are required to diffusive toward the alternating layers of the BCP. Finally, the slowest kinetics is required for the transition due to effective solvent changes for ψ̅ = 0.25, *ϕ*_*p*_ = 0.1 and *ψ*_0_ = −0.5.

**Figure 11:**
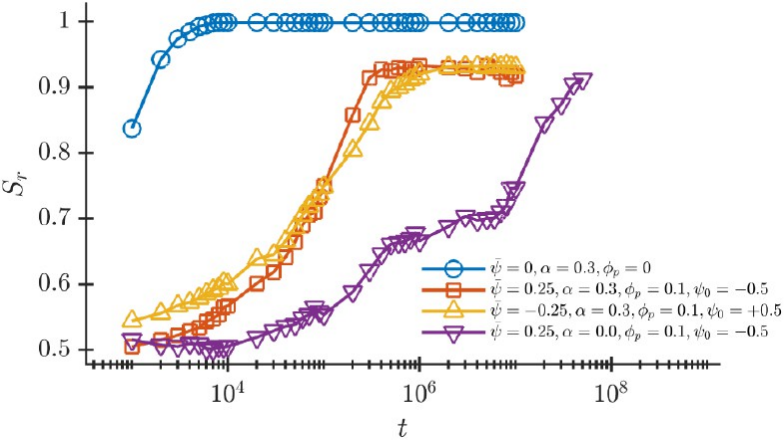
Kinetics of the onion phase in the presence of NPs with different affinity *ψ*_0_ for various solvent selectivities *α* and with the neat BCP onion phase (*ϕ*_*p*_ = 0, *α* = 0.3, ψ̅ = 0) as baseline. Each curve is an average over 20 independent runs.

So far we have assumed that NPs have a strong affinity toward either of the monomers composing the BCP chain. Figure 12 shows the phase behavior of a BCP droplet with composition ψ̅ in a selective solvent with *α* = 0.3 in the presence of a concentration of *ϕ*_*p*_ of NPs with affinity *ψ*_0_ = 0, indicating that they are surfactant-like NPs that segregate toward the interface in a BCP melt. At low concentrations, they are shown to weakly modify the BCP morphology. Importantly, they roughly preserve the onion-like morphology of the BCP droplet for ψ̅ ∼ 0. For higher concentration, NPs can significantly impact the overall effective incompatibility between A and B monomers, reducing the effective *χ* *N* parameter leading to large homogeneous regions where the BCP is approximately homogeneous. For ψ̅ = 0 and *ϕ*_*p*_ = 0.1 a large mixed region can be

**Figure 12:**
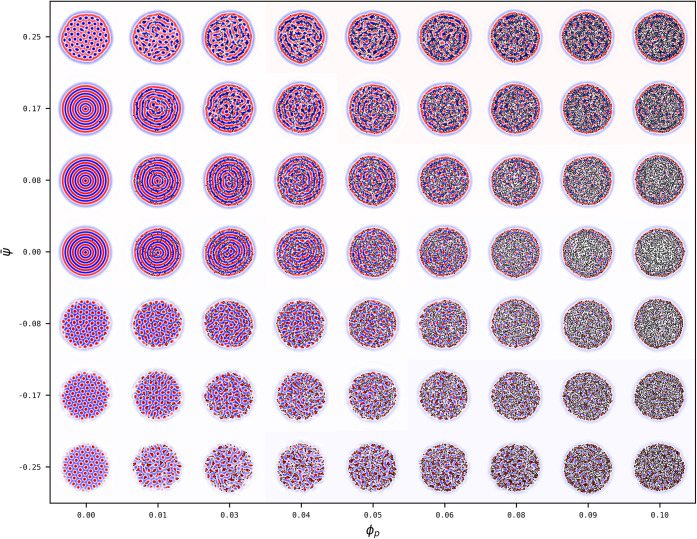
Phase diagram of BCP droplets with composition ψ̅ in a selective solvent with *α* = 0.3 in the presence of a concentration *ϕ*_*p*_ and NP affinity *ψ*_0_ = +0.0. The same color code is used as in Fig. 5.

observed (in white), surrounded by a ring of A monomers due to the *α* = 0.3 selectivity of the solvent.

## Conclusions

This work has presented a model that combines a two-order-parameter^31,35^ description of the BCP blend, along with a hybrid particle/field scheme^46^ to describe nanocomposites, to study the phase behavior of BCP droplets and BCP droplets in the presence of NP additives. The model reproduces several experimental and theoretical results, and predicts the phase diagram of this complex hybrid system under various conditions, at a modest computational cost. More sophisticated models such as self-consistent field theory or molecular dynamics^58^ can exploit the results shown in this work, by directly focusing on specific regimes delineated in this work. Furthermore, future work can explore 3D systems using the same hybrid model as presented here and focusing on the emergent onion phases due to the presence of NPs.

By systematically studying the phase behavior of pure BCP blends under various composition and solubility conditions, it has been possible to identify a mixed morphology consisting of half-and-half hexatic circular and onion-like phase, spatially segregated within the BCP droplet bulk. This Janus-like morphology can be obtained as an intermediate phase when exploring solvent solubility at constant BCP composition, in a similar way as the onion-and-stripe morphology which is well reported in experiments^19^ and simulations.^31^

The morphology of BCP droplets under soft confinement in the presence of NPs with various solubility conditions and BCP compositions has been systematically explored by means of morphological phase diagrams.

NPs have been found to hierarchically segregate within BCP-rich droplets. Furthermore, they are found within their preferential BCP domains, according to their solubility, following various experimental results.^15,16,19^ Several order-to-order transitions have been identified due to the presence of NPs that can modify the effective BCP composition.

Two onion-to-sphere morphological transitions have been identified -see Figures 8 and 10 for ψ̅ ∼ 0- in accordance with experimental results by Yabu et al.^50^ Effective changes in the BCP droplet solubility with respect to the solvent can trigger additional phase transitions: a hexatic-to-ellipsoid-to-onion phase transition has been identified at moderate NP loads with NPs miscible in the minority domain in a neutral solvent.

## Supplementary Material



## Notes

Quench from disordered morphology; phase behavior of BCP droplet; phase diagram of the BCP/solvent system in terms of the BCP composition (PDF)
