# Supplementary material for: Block Copolymer Nanocomposites under Soft Confinement
Source: Macromolecules. 2025 May 14;58(10):5240–53. doi: 10.1021/acs.macromol.4c03184 (PMC12121502; doi:10.1021/acs.macromol.4c03184)
Supplement: Supplementary file 1 [file ma4c03184_si_001.pdf]

Supporting Information to: Block copolymer nanocomposites under soft confinement

Javier Diaz*[1,2], Marco Pinna†[3], Andrei Zvelindovsky‡[3], and Ignacio Pagonabarraga§[1,2]

[1]Departament de Física de la Matèria Condensada, Universitat de Barcelona, Martí i Franqués 1, 08028 Barcelona, Spain

[2]Universitat de Barcelona Institute of Complex Systems (UBICS), Universitat de Barcelona, 08028 Barcelona, Spain

[3]School of Engineering and Physical Sciences, College of Health and Science, Center for Computational Physics, University of Lincoln, Brayford Pool, Lincoln, LN6 7TS, UK

May 7, 2025

1 Quench from disordered morphology

To justify the use of an already phase separated ϕ configuration at $t = 0$, we report the phase diagram of a pure BCP/solvent blend ($\phi_p = 0$) in Fig. S1 for $R_{drop}/H_0 = 2.25$, which should be compared with Fig. 1 (a). Here, we consider a quench from a completely disordered system and the initial droplet is not prescribed. One may observe that the BCP droplet morphology is the same irregardless of the initial condition.

2 Phase behavior of BCP droplet for $R_{drop}/H_0 = 9$

3 Detail of phase diagram in Fig. 1 (b)

*jdiazbranas@ub.edu
†mpinna@lincoln.ac.uk
‡AZvelindovsky@lincoln.ac.uk
§ipagonabarraga@ub.edu

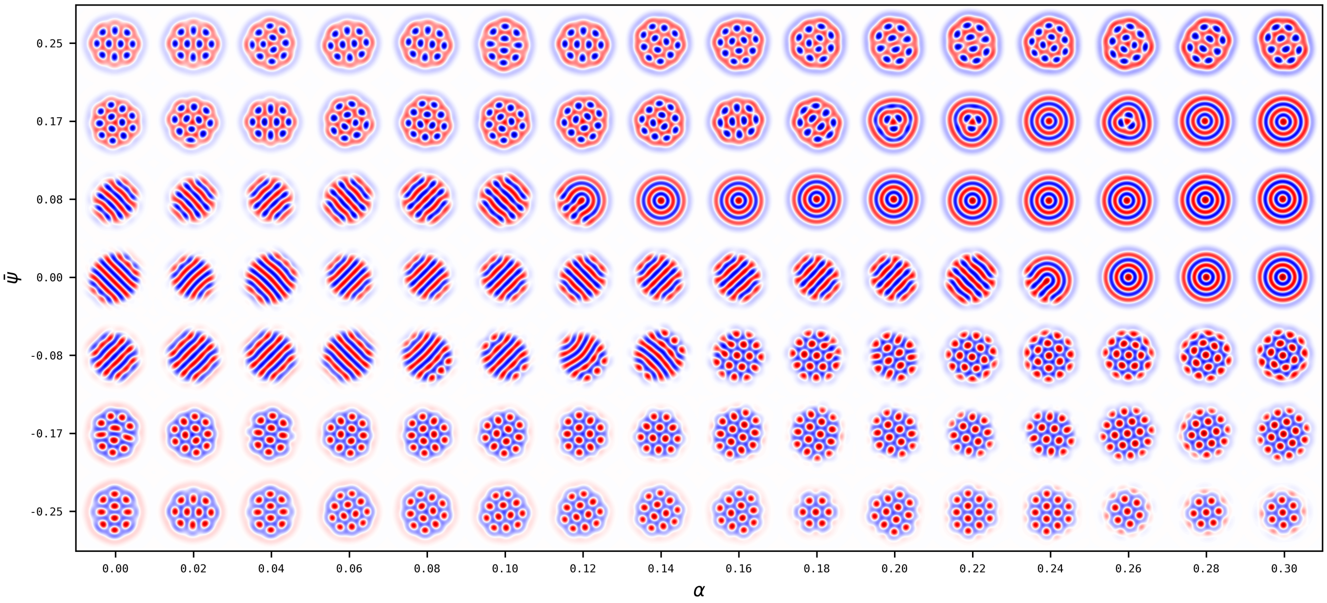

Figure S1: Phase diagram of the BCP/solvent system in terms of the BCP composition $\bar{\psi}$ and the solubility α with a completely random initial condition at $t = 0$, with droplet size $R_{drop}/H_0 = 2.25$. Each snapshot is centered at the center of mass of the final macroscopic droplet.

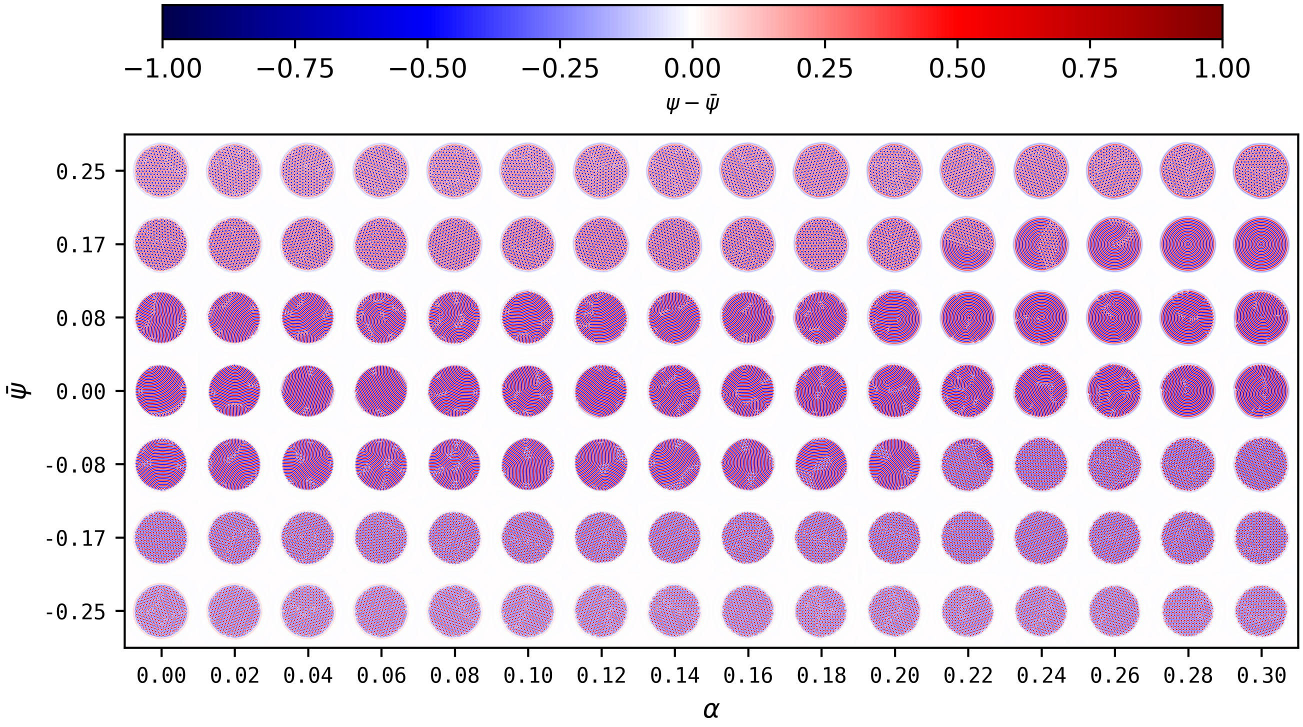

Figure S2: Phase diagram of the BCP/solvent system in terms of the BCP composition $\bar{\psi}$ and the selectivity α. For each point $(\alpha,\bar{\psi})$, a snapshot of the system is shown as the colormap of the field $\psi - \bar{\psi}$. The BCP/solvent fraction is kept constant at $\bar{\phi} = 0.5$ while the system size is $L_x = L_y = 256$ leading to a droplet size $R_{drop}/H_0 = 9.0$.

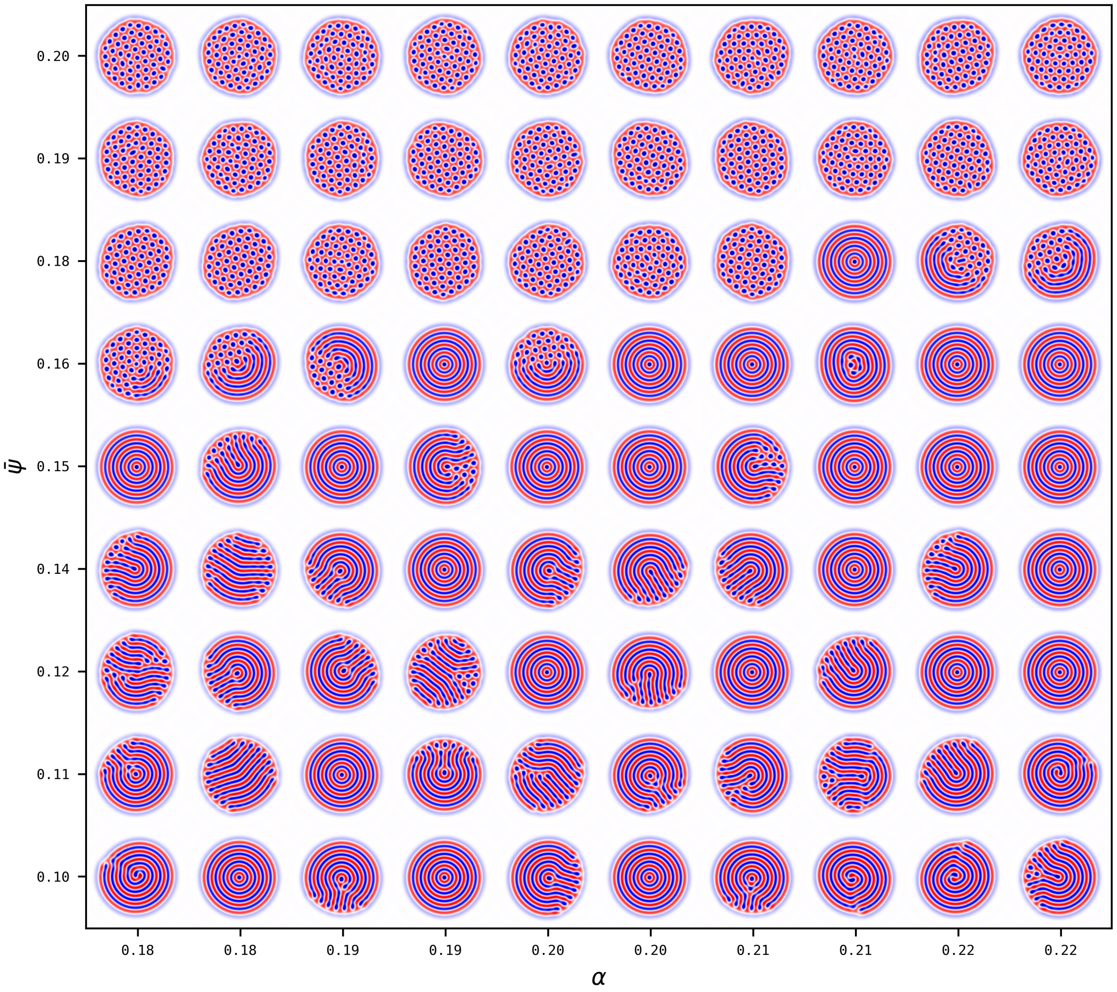

Figure S3: Detail of the phase diagram shown in Fig. 1 (b)
